# The Defense-Related Isoleucic Acid Differentially Accumulates in *Arabidopsis* Among Branched-Chain Amino Acid-Related 2-Hydroxy Carboxylic Acids

**DOI:** 10.3389/fpls.2018.00766

**Published:** 2018-06-08

**Authors:** Rafał P. Maksym, Andrea Ghirardo, Wei Zhang, Veronica von Saint Paul, Birgit Lange, Birgit Geist, Mohammad-Reza Hajirezaei, Jörg-Peter Schnitzler, Anton R. Schäffner

**Affiliations:** ^1^Institute of Biochemical Plant Pathology, Helmholtz Zentrum München, Munich, Germany; ^2^Research Unit for Environmental Simulation, Helmholtz Zentrum München, Munich, Germany; ^3^Molecular Plant Nutrition, Leibniz-Institute for Plant Genetics and Crop Plant Research, Gatersleben, Germany

**Keywords:** isoleucic acid, branched-chain amino acids, *Arabidopsis thaliana*, plant pathogen defense, *UGT76B1*, *Pseudomonas syringae*, GC–MS, MSUD

## Abstract

The branched-chain amino acid (BCAA) related 2-hydroxy carboxylic acid isoleucic acid (ILA) enhances salicylic acid-mediated pathogen defense in *Arabidopsis thaliana*. ILA has been identified in *A. thaliana* as its glucose conjugate correlated with the activity of the small-molecule glucosyltransferase UGT76B1, which can glucosylate both salicylic acid and ILA *in vitro*. However, endogenous levels of the ILA aglycon have not yet been determined *in planta*. To quantify ILA as well as the related leucic acid (LA) and valic acid (VA) in plant extracts, a sensitive method based on the derivatization of small carboxylic acids by silylation and gas chromatography–mass spectrometric analysis was developed. ILA was present in all species tested including several monocotyledonous and dicotyledonous plants as well as broadleaf and coniferous trees, whereas LA and VA were only detectable in a few species. In *A. thaliana* both ILA and LA were found. However, their levels varied during plant growth and in root vs. leaves. ILA levels were higher in 2-week-old leaves and decreased in older plants, whereas LA exhibited a reverted accumulation pattern. Roots displayed higher ILA and LA levels compared to leaves. ILA was inversely related to *UGT76B1* expression level indicating that UGT76B1 glucosylates ILA *in planta*. In contrast, LA was not affected by the expression of *UGT76B1.* To address the relation of both 2-hydroxy acids to plant defense, we studied ILA and LA levels upon infection by *Pseudomonas syringae.* LA abundance remained unaffected, whereas ILA was reduced. This change suggests an ILA-related attenuation of the salicylic acid response. Collectively, the BCAA-related ILA and LA differentially accumulated in *Arabidopsis*, supporting a specific role and regulation of the defense-modulating small-molecule ILA among these 2-hydroxy acids. The new sensitive method will pave the way to further unravel their role in plants.

## Introduction

Plants have evolved an array of different defense mechanisms to cope with diverse biotic and abiotic stress factors. Plant immune responses are activated upon recognition of PAMPs or virulence effectors, activating PTI or ETI, respectively (Jones and Dangl, 2006; Spoel and Dong, 2012). The need to fine-tune defense responses requires a plethora of downstream responses to PTI and ETI, which are tightly regulated by distinct signaling pathways depending on the attacker’s life style. The SA-dependent signaling pathway primarily mediates the response against biotrophic and (hemi-) biotrophic pathogens, whereas the JA-signaling pathway is required for resistance against necrotrophic pathogens. The interaction between SA- and JA-signaling is mostly antagonistic, and both pathways are essential for modulating plant defense response to biotic stresses (Pieterse et al., 2009, 2013; Vlot et al., 2009; Dodds and Rathjen, 2010). However, during the past few years several new compounds have been identified and linked to the regulation of plant defense response. Several of these are amino acid-related molecules like pipecolic acid derived from lysine or ILA, which is thought to be linked to isoleucine metabolism (Yasuda, 2007; von Saint Paul et al., 2011; Návarová et al., 2012; Vogel-Adghough et al., 2013; Zeier, 2013; Aranega-Bou et al., 2014; Yang and Ludewig, 2014; Gao et al., 2015; Baccelli and Mauch-Mani, 2016; Bernsdorff et al., 2016).

Recent studies have demonstrated that the abundances of the BCAAs Val, Leu, and Ile increase upon *Pseudomonas syringae* infection in *Arabidopsis thaliana* (Návarová et al., 2012) and *Nicotiana tabaccum* (Vogel-Adghough et al., 2013), suggesting that BCAAs or molecules related to BCAAs metabolism might be involved in plant defense. Importantly, the potentially BCAA-related 2-hydroxy carboxylic acid (2-HA) ILA (C_6_H_12_O_3_; 2-hydroxy-3-methylpentanoic acid) could modulate the SA-JA crosstalk in *A. thaliana* (von Saint Paul et al., 2011). In humans suffering from MSUD, ILA and the related 2-HAs VA (C_5_H_10_O_3_; 2-hydroxy-3-methylbutyric acid) and LA (C_6_H_12_O_3_; 2-hydroxyisocaproic acid) highly accumulate. They originate from the chemical reduction of enhanced levels of the corresponding 2-keto acids, which are the primary degradation products of the BCAAs Ile, Val, and Leu, respectively. In MSUD patients the levels of these 2-keto acids rise due to various mutations of the branched chain 2-ketoacid dehydrogenase complex catalyzing the subsequent step in their catabolism (Liebich and Först, 1984; Mamer and Reimer, 1992; Podebrad et al., 1997).

In plants, ILA could be associated to plant defense through the functional study of the small-molecule glucosyltransferase UGT76B1. Plants contain a large number of *UGT* genes, which utilize UDP-activated carbohydrates to glycosylate a plethora of small molecules such as signaling molecules or secondary metabolites (Bowles et al., 2006). The broadly stress-inducible family member UGT76B1 was shown to impact the SA- and JA-mediated defense pathways and to induce JA-related responses. A *ugt76b1* loss-of function mutant showed enhanced resistance to the biotrophic pathogen *P. syringae*, whereas a constitutive *UGT76B1* overexpression line became more susceptible compared to wild-type plants. Infections with the necrotrophic pathogen *Alternaria brassicicola* revealed the opposite resistance phenotype in agreement to the antagonistic behavior of the SA- and JA-signaling pathways (von Saint Paul et al., 2011). UGT76B1 was related to two aglyca in *A. thaliana*. Noutoshi et al. (2012) demonstrated its ability to glucosylate SA; however it was also conjugating ILA, which in turn inhibited the glucosylation of SA (von Saint Paul et al., 2011; Noutoshi et al., 2012). In a non-targeted metabolome analysis, ILA glucoside levels were correlated with UGT76B1 expression; they were low in *ugt76b1* loss-of-function mutants and enhanced in lines constitutively expressing UGT76B1. Exogenous application of ILA was able to enhance defense responses. However, the aglycon ILA itself was not detected in that study (von Saint Paul et al., 2011). *In planta*, the further elucidation of the biological role and the biosynthesis of ILA was hampered by the lack of a sensitive method to detect the 2-HAs, which accordingly do not appear in plant metabolome databases (e.g., KNApSAck database^1^).

Therefore, here we aimed to develop a sensitive method to quantify ILA and simultaneously two other BCAA-related 2-HAs, LA and VA, in plant extracts based on silylation and gas chromatography–mass spectrometry (GC–MS). We show that ILA is present in all plant species analyzed, whereas VA and LA are species-dependent. In *A. thaliana* ILA and LA were present, but accumulated differentially in root and shoots, during plant development and upon infection with the pathogen *P. syringae*.

## Materials and Methods

### Chemicals

Isoleucic acid [(2S, 3S)-2-hydroxy-3-methylpentanoic acid] was purchased from Interchim (Montluçon, France). Leucic acid (2-hydroxyisocaproic acid), VA [(S)-(+)-2-hydroxy-3-methylbutyric acid], 2-hydroxyhexanoic acid and 4-nitrophenol were obtained from Sigma-Aldrich (Munich, Germany). The derivatization reagent BSTFA containing 1% TMCS was purchased from Macherey-Nagel (Düren, Germany). All other chemicals were of the highest commercially available grades.

### Plant Materials and Growth Conditions

*Arabidopsis thaliana* ecotype Col-0 was the genetic background of wild-type plants. The loss-of-function mutant of the UDP-glucosyltransferase gene *ugt76b1-1* (Col-0) and overexpression line *UGT76B1-OE7* were previously described (von Saint Paul et al., 2011). Rosette leaves for metabolite extraction were harvested from *Arabidopsis* plants grown on peat moss-based soil (Floragard Floradur B fein; Floragard, Surberg, Germany) mixed with quartz sand (8:1) at a photoperiod of 10 h and light intensities [incident photosynthetically active photon flux density (PPFD)] of 180 μmol m^-2^ s^-1^, at 20/18°C (day/night) and 75% relative humidity. For the analysis of the 2-HAs ILA, LA, and VA in roots of *Arabidopsis*, plants were grown on square plates containing ½ Murashige-Skoog medium with vitamins [1% sucrose; 0.5% (w/v) Gelrite (Duchefa, Haarlem, Netherlands)] and grown vertically under the same conditions. Barley (*Hordeum vulgare*), tabacco (*N. tabaccum*) and *Brassica nigra* were grown on soil under the same environmental condition as *Arabidopsis*, but at 60% relative air humidity. Tomato (*Solanum lycopersicum*) plants were grown at 29/18°C and 54/72% relative air humidity (day/night). In all cases leaves were sampled for metabolite extraction. Tree samples were grown under different conditions, optimal for each species. Silver birch (*Betula pendula*), Scots pine (*Pinus sylvestris*), and European larch (*Larix decidua*) were 2-year-old plants and were grown under field condition of summer temperature and light of 10–30°C and 100–1500 μmol m^-2^ s^-1^ (Ghirardo et al., 2010). Leaf material was collected after acclimating plants to 30°C and 1000 μmol m^-2^ s^-1^ PPFD. Grafted oak (*Quercus robur*) trees originated from a natural oak forest in North Rhine-Westphalia, Germany; leaves of oak trees with a tolerant phenotype to the herbivore *Tortrix viridana* from the population ‘Asbeck’ were used (Ghirardo et al., 2012). Wild-type gray poplar (*Populus x canescens*) saplings were 3-year-old (Ghirardo et al., 2014) and were grown and cultivated according to Behnke et al. (2007) and Cinege et al. (2009). Poplar samples were collected at 27°C and ∼500 μmol m^-2^ s^-1^ PPFD. The whole rosettes or roots were used to determine the abundance of VA, LA, and ILA (next section) in plant tissues of *Arabidopsis* as well as in leaves and needles for the other plant species.

### Determination of ILA, LA, and VA by GC–MS

Plant materials were harvested by flash-freezing in liquid N_2_, followed by 24 h lyophilization. Twenty mg of a dried plant material was transferred to the grinding tubes (Ceramic beads for cell lysis, Genaxxon, Ulm, Germany) and homogenized in a MP FastPrep-24 Homogenizer (MP Biochemicals, Heidelberg, Germany) for 2 min. Before use, grinding tubes were conditioned twice with hexane (Sigma-Aldrich, Munich, Germany) and twice with HPLC-grade water (Merck, Darmstadt, Germany) and dried *in vacuo*. Metabolites were extracted with 1 mL 70/30% methanol/H_2_O (v/v) (pre-cooled to 4°C) containing the first IS, 2-hydroxyhexanoic acid (Sigma-Aldrich, Munich, Germany; 2.5 mg L^-1^) to control the extraction procedure. Solvent was added directly into the grinding tubes, and samples were extracted by shacking tubes for 60 min at 4°C. The extraction solution was centrifuged for 15 min at 18,000 *g*-force and 4°C, the supernatant was transferred into a fresh 2 mL collection tube and re-centrifuged for 10 min. Nine-hundred μL of the supernatant was transferred into a fresh 2 mL plastic tube. Extracts were dried in a speedvac concentrator and dissolved in 1 mL of 25 mM ammonium acetate (pH 6–7). To fully dissolve the sample, suspension was sonicated for 3 min at 50% power (Branson Sonifier Cell Disruptor B15, Branson Ultraschall, Dietzenbach, Germany) and incubated on shaker for 5 min at 4°C. Extracts were purified on SPE weak anion exchange columns (StrataX-AW 30 mg mL^-1^, Phenomenex, Aschaffenburg, Germany). Prior loading the sample, the SPE columns were conditioned with 0.5 mL methanol and equilibrated with 0.5 mL HPLC-grade water (Merck, Darmstadt, Germany). After applying the samples, two washing steps of 0.5 mL 25 mM ammonium acetate and 0.5 mL methanol were performed. Metabolites were eluted twice with 0.5 mL methanol containing 5% (v/v) formic acid. Samples were dried and dissolved in 200 μL methanol containing a second IS 4-nitrophenol (Sigma-Aldrich, Munich, Germany; 12.5 mg L^-1^) and transferred to 250 μL glass inserts (Sigma-Aldrich, Munich, Germany). Samples were dried, inserts were transferred into amber glass vials, derivatized for 120 min at 60°C using 50 μL BSTFA containing 1% TMCS.

Qualitative and quantitative analyses of VA, LA, and ILA were performed by GC–MS. Samples were analyzed with a thermo-desorption unit (Gerstel, Mülheim, Germany) coupled to a GC–MS instrument (GC type: 7890; MS type: 5975C, both Agilent Technologies, Palo Alto, CA, United States). The thermo-desorption unit was used as injector for the conversion of the sample from liquid to gas-phase. GC–MS was run as follows: 1 μL of sample was injected into the thermo-desorption unit in a dedicated glass tube containing the glass insert for liquid injection (both from Gerstel, Mülheim, Germany). Prior to each analysis, tubes and inserts were accurately cleaned with acetone, methanol and water, separately used in ultrasonic bath for 30 min each, and kept in hexane solution overnight. Immediately before analysis, tubes were baked out in oven at 400°C for 1 h under ∼80 mL min^-1^ N_2_ (5.0 gas purity) flow. Samples were vaporized by quickly rising the temperature from 40 to 270°C at a rate of 360°C min^-1^ and holding for 0.5 min. The compounds were refocused using a Cryo Injection System (Gerstel, Mülheim, Germany) at -50°C, then desorbed and injected in splitless mode by rising the temperature to 250°C at a rate of 12°C s^-1^ and hold for 1.5 min, followed by ramping at 12°C s^-1^ to 275°C and holding for 2 min. Separation was achieved by using the Agilent J&W HP-5 ms GC column (30 m × 250 μm × 0.25 μm) with 1 mL min^-1^ constant flow rate of He, and a temperature program of 90°C for 4 min, followed by ramping at 2°C min^-1^ to 120°C and holding for 0 min, then 100°C min^-1^ to 300°C and holding for 5 min. Identification of VA, LA, ILA and the two IS (2-hydroxyhexanoic acid and 4-nitrophenol) were achieved by spectra and retention time comparison of pure standards. The quantification was obtained by means of a calibration curve obtained from pure standards. MS spectra were parallelly acquired in total ion current and in selective ion monitoring modes. Scans of total ion current were performed in the range of 35–300 mass-to-charge ratios (m/z) (threshold: 150; 7.76 scan/s^-1^). Selective ion monitoring parameters were as follows, VA: start time: 6.2 min, ion: 147.0 m/z, dwell: 150 ms; LA: start time: 8.5 min, ion: 145.0 m/z, dwell: 150 ms; ILA: start time: 11.5 min, ion: 159.0 m/z, dwell: 150 ms; 2-hydroxyhexanoic acid: start time: 13.9 min, ion: 173.1 m/z, dwell: 100 ms; 4-nitrophenol: start time: 16.0 min, ion: 196.1 m/z, dwell: 25 ms. MS detector was kept off until 6.20 min and switched off after 20.65 min until the end of the run. Calibration was achieved by adding 0, 0.05, 0.1, 0.15, 0.2, 0.25, 0.3, 0.5, 1, 5, 10 mg L^-1^ of ILA into aliquots of the same (pooled)plant extract in order to take into account potentially occurring matrix effects. Each concentration of ILA contained the same fix concentration of IS (50 mg L^-1^). Calibration samples were treated in exactly the same way as the sample preparation explained above (i.e., passing through the SPE columns). Data were background corrected using the mean value obtained from measuring the plant extract with the addition of the standard solution lacking ILA to correct for the basal levels of ILA present in the pooled plant material. Standards were prepared independently in triplicate, and each concentration was measured twice. The two technical replicates were averaged and their means were further used for the calculation of response factors. To consider uncertainties of standard preparation, the quantification of 2-HAs were based on three independently created serial dilutions. The resulting MS signal responses were found to be linear (*R*^2^ > 0.9999) with an increasing standard concentration. Response factors of VA and LA were calculated based on the matrix-dependent calibration curve of ILA assuming that the matrix effects occurred at the same extent for VA, LA, and ILA: serial dilutions of pure standards (0–100 mg L^-1^) of ILA, VA, and LA were measured in parallel and the ratios of VA/ILA and LA/ILA were applied to the matrix-dependent response factor of ILA. Data were always normalized to IS values of 4-nitrophenol. Samples showing an inconsistent ratio of the two IS were discarded from the analysis. Limits of detection were calculated using 2 sigma (σ) and were ranging between 0.027–1.29 (ILA), 0.045–0.229 (VA), 0.012–0.029 (LA) ng g^-1^ DW, referred to *A. thaliana* plant material. The limits of quantification (LOQ) were set to three times of their respective limits of detection.

### *In Vitro* Analysis of the Activity of UGT6B1 Toward ILA, LA, and SA

UGT76B1 recombinant fusion protein harboring an N-terminal GST was produced according Messner et al. (2003). The coding region of *UGT76B1* including its 5′ ATG codon was cloned into pDEST15 (Invitrogen, Germany). To analyze the activity of UGT76B1 toward ILA and LA, UGT76B1 recombinant protein was incubated with UDP-glucose and either with ILA or with LA. The reaction mixture was composed of 0.1 M Tris-HCl buffer pH 7.5, 2 mM ^14^C-UDP-glucose (37 Bq nmol^-1^; Hartmann Analytics, Germany), 0, 0.1, 0.2, 0.4, 0.6, 0.8, 1.0, 1.5, and 2.0 mM aglycon, and 0.5 μg recombinant protein in 50 μL. In case of SA, 0.05, 0.1, 0.2, 0.3, 0.4, 0.5, 0.6, 0.8, 1.0, 1.5 mM aglycon and 2 mM UDP-glucose was used. The reaction mixture was incubated for 30 min at 30°C in a water bath and stopped by adding 15 μL 0.5 M phosphoric acid. The reaction mixture was centrifuged and 40 μL of the supernatant separated by HPLC coupled with radiodetection (Messner et al., 2003; Ramona Star, Elysia-raytest, Straubenhardt, Germany). The amount of the formed glucosylated products were calculated from the separated product peak relative to the whole radioactivity in each HPLC run in case of reactions with ILA and LA. In case of SA as substrate, the formation of SA-*O*-glucoside was quantified based on UV absorption at 302 nm and a standard curve derived by enzymatically prepared SA-*O*-glucoside. Enzyme parameters (*k*_cat_, *K*_m_) were obtained via non-linear regression using GraphPad Prism version 6.07 (GraphPad Software, La Jolla, CA, United States).

### Infection of *A. thaliana* With *Pseudomonas syringae*

*Pseudomonas syringae* pv. *tomato* DC3000 (virulent strain) and *P. syringae* pv. *tomato* DC3000 (avrRpm1) (avirulent strain) were used to test the effects of biotic stress on endogenous levels of ILA and LA. Bacteria from -80°C glycerol stocks were streaked out onto solid NYGA medium (Daniels et al., 1984) and grown for 2 days at 28°C. A single colony was picked and transferred to liquid NYG medium containing antibiotics (50 mg L^-1^ kanamycin and 50 mg L^-1^ rifampicin) and grown overnight at 28°C with shaking at 170 rpm. Bacteria were grown until the late log phase (OD_600_ 0.6 to 1.0). Plants were infected by spraying with bacteria diluted to OD_600_ = 0.2 with 10 mM MgCl_2_ containing 0.01% Silwet-77. OD_600_ = 0.2 corresponds to approximately 1 × 10^8^ colony-forming units mL^-1^. Control solutions (mock) did not contain bacteria. The whole rosettes were harvested 24 h post-infection and stored at -80°C.

### Determination of BCAAs in *A. thaliana*

Branched-chain amino acids were extracted and analyzed essentially as described by Höller et al. (2014) except for the separation of amino acids carried out by UPLC (AcQuity H-Class, Waters) on a C18 reversed phase column (ACCQ-Tag UltraC18, 1.7 μM, 2.1 mm × 100 mm) at 45°C with a flow rate of 0.7 mL min^-1^, run time of 10.2 min, excitation at 266 nm and detection at 473 nm (Hilo et al., 2017). BCAA determination after spraying 1 mM of ILA, LA, or VA onto the rosette leaves of 4-week-old *A. thaliana* plants and further cultivation for 24 h was performed by GC–MS at the Faculty of Biology of the Ludwig-Maximilians-Universität München as described previously (Georgii et al., 2017).

### Statistical Analysis

For statistical analysis, the software suites SigmaPlot v11.0 (Systat Software, Erkrath, Germany) and R^2^ were used. Two-way ANOVA followed by one-sided Dunnett *post hoc* multiple comparison test was employed to test significant changes of 2-HAs among genotypes and during plant development, using as control of the statistical test the wild-type genotype and 2-week-old plants, respectively. When the data did not follow a normal distribution, Kruskal–Wallis was used and indicated in the text. Developmental changes of BCAAs as well as ILA and LA levels following pathogen infection were tested using Student’s *t*-test (SigmaPlot).

## Results

### Determination of BCAA-Related 2-HAs in Plants

**FIGURE 1:**
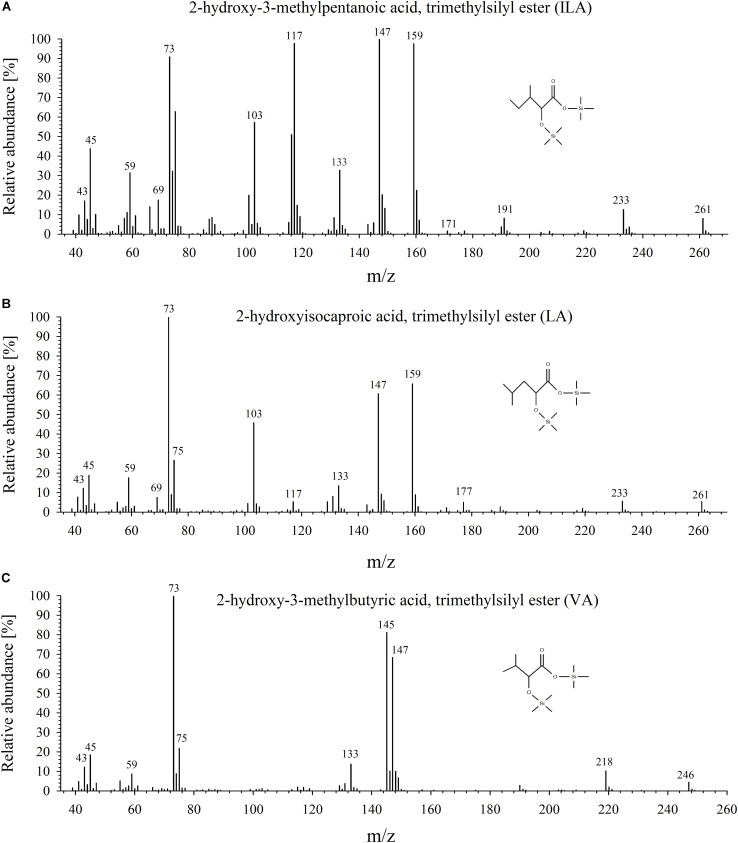
Mass-spectrometric analysis of ILA, LA, and VA. Mass spectra of the trimethylsilyl-derivatized products for **(A)** ILA, **(B)** LA, and **(C)** VA.

**FIGURE 2:**
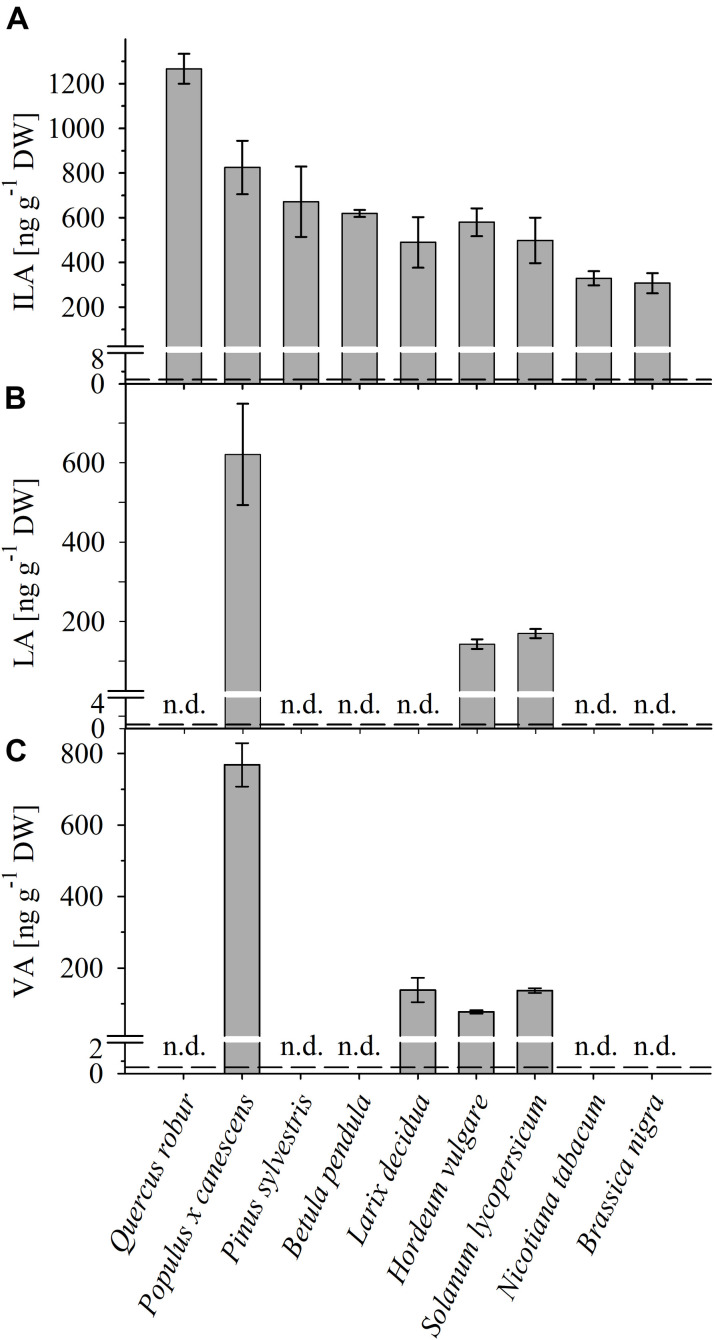
Detection of ILA, LA, VA in different plant species. Abundance of **(A)** ILA, **(B)** LA, and **(C)** VA in leaf extracts of monocot (*Hordeum vulgare*) and dicot crops (*Solanum lycopersicum, Nicotiana tabacum, Brassica nigra*), as well as of broadleaf (*Quercus robur, Populus x canescens, Betula pendula*) and coniferous (*Pinus sylvestris, Larix decidua*) trees. Means ± SE (*n* = 3–4) are plotted; n.d., not detected. Dashed lines indicate the limit of detection (see section “Materials and Methods”).

**FIGURE 3:**
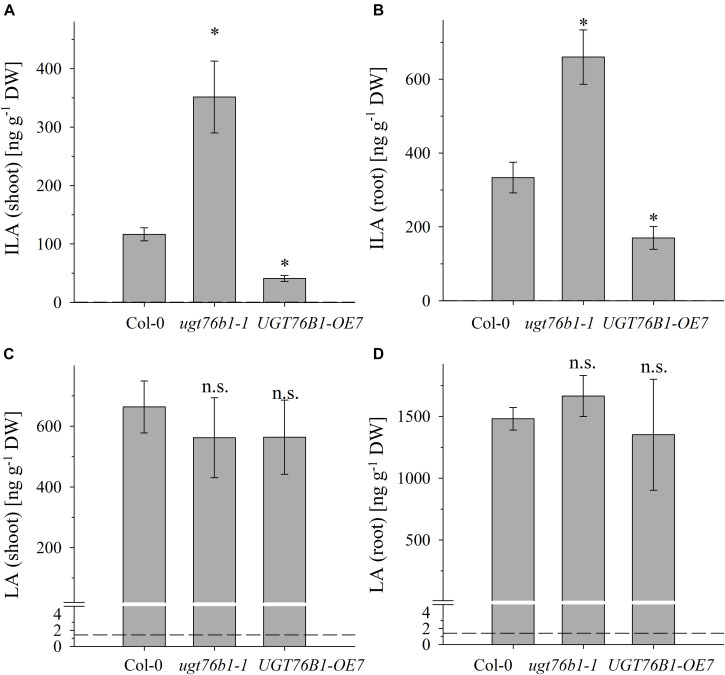
The abundance of ILA and LA in above- and below-ground tissues of Col-0, *ugt76b1-1* and *UGT76B1-OE7.* Levels of **(A,B)** ILA and **(C,D)** LA in **(A,C)** shoot and in **(B,D)** root in 3-week-old Col-0, *ugt76b1-1* and *UGT76B1-OE7* plants grown on Gelrite plates in short-day conditions (10 h photoperiod). Means ± SE (*n* = 4) are plotted. Asterisks indicate statistically significant differences in comparison to wild type (Col-0) (*ANOVA*), ^∗^*p*-value < 0.05. Dashed lines indicate the limit of detection (see section “Materials and Methods”).

To identify and quantify the abundance of VA (2-hydroxy-isovaleric acid), LA (2-hydroxyisocaproic acid) and ILA (2-hydroxy-3-methyl-valeric acid) in plants, we developed a sensitive method based on derivatization of these molecules by silylation and GC–MS analysis (**Figure 1** and Supplementary Figure S1). With this method it was possible to detect low amounts of the BCAA-related compounds. The limits of detection were ranging between 0.027–1.29 (ILA), 0.045–0.229 (VA), 0.012–0.029 (LA) ng g^-1^ DW depending on instrument performance and background noise. To demonstrate the general versatility of the procedure different plant species were examined for their content in ILA, LA, and VA. ILA could be ubiquitously detected in all examined plant species including monocotyledonous and dicotyledonous plants, herbaceous and woody plants, whereas VA and LA were found only in some species (**Figure 2** and Supplementary Figures S2, S3). All three 2-HAs could be detected in *Populus x canescens, Hordeum vulgare*, and *Solanum lycopersicum* (**Figure 1**). In the model plant *A. thaliana* only LA and ILA were present in the extracts (**Figure 3** and Supplementary Figure S3).

### UGT76B1 Affects the Accumulation of ILA in *Arabidopsis thaliana*

We previously correlated the abundance of ILA glucoside in leaves with the level of *UGT76B1* expression in loss-of-function mutants and transgenic overexpression lines (von Saint Paul et al., 2011). The method to quantify ILA and to distinguish it from the isomeric LA now allowed elucidating the impact of the UGT76B1 level on the abundance of the ILA aglycon as well. We quantified ILA in the loss-of-function mutant *ugt76b1-1*, in the corresponding wild-type Col-0 and in the constitutive overexpression line *UGT76B1-OE7* as a function of the plant developmental stage in rosette leaves and roots of plate grown *A. thaliana* seedlings. Independent of the genotypes, ILA and LA were more abundant in roots. With respect to the genotypes, elevated levels of ILA aglycon were detected in *ugt76b1-1*, whereas decreased levels were found in *UGT76B1-OE7* in both shoot and root tissues (**Figures 3A,B**). Thus the amount of ILA aglycon was inversely related with the expression levels of *UGT76B1* in the different genotypes opposite to ILA glucoside as shown previously, suggesting ILA as an *in vivo* substrate of UGT76B1.

In contrast to ILA, the abundance of LA was not affected by the expression levels of *UGT76B1* in the different genotypes (**Figures 3C,D**). This finding is surprising at first glance and may suggest that UGT76B1 is not acting on LA *in vivo* or that the level of LA is independently regulated and not affected by UGT76B1. Recombinantly expressed UGT76B1 is able to glucosylate both ILA and LA *in vitro*, which is in line with the highly similar structure of the two isomers. While *K*_m_ values were almost identical for both substrates, *k*_cat_ for ILA as a substrate was about 50% higher compared to LA (**Table 1** and Supplementary Figure S4). The UGT76B1 substrate SA had both a lower *K*_m_ and *k*_cat_ value than ILA resulting in a similar enzymatic efficiency *k*_cat_/*K*_m_ for both substrates (**Table 1**).

**Table 1:** Enzyme activities of recombinant UGT76B1 toward ILA, LA, or SA.

	ILA	LA	SA
*K*_m_ [mM]	0.472 ± 0.097	0.461 ± 0.079	0.151 ± 0.028
*k*_cat_ [s^-1^]	0.914 ± 0.067	0.582 ± 0.035	0.331 ± 0.016
*k*_cat_/*k*_m_ [s^-1^ mM^-1^]	1.94	1.26	2.2

*Enzyme reactions were performed using recombinantly expressed UGT76B1 and ^14^C-labeled UDP-glucose together with ILA or LA as aglycon substrates to obtain k_cat_ and K_m_ values (see section “Materials and Methods”; n = 4, ±SE). Values with SA were determined based on the absorption of SA and SA glucoside at 302 nm (see section “Materials and Methods”; n = 3, ±SE). While K_m_ values for ILA and LA were similar, k_cat_ were significantly different (t-test, p = 0.005). The derived enzymatic efficiencies k_cat_/K_m_ are indicated as well.*

### ILA and LA Are Differently Affected During Growth and Development

**FIGURE 4:**
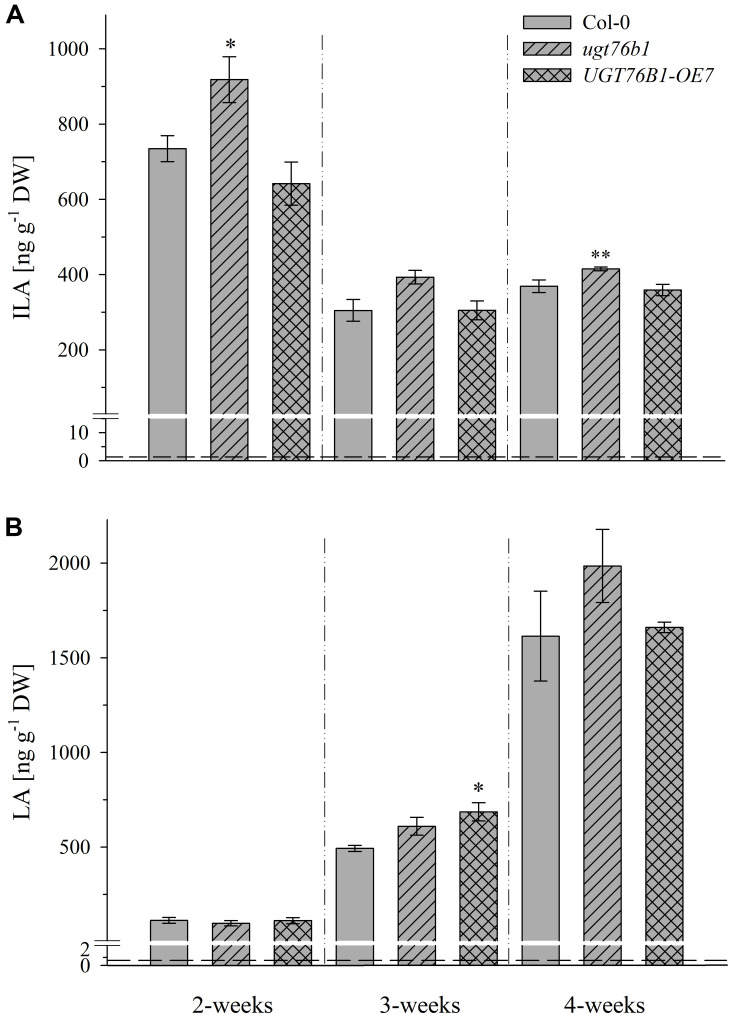
Isoleucic acid and LA abundance in different developmental stages. Levels of **(A)** ILA and **(B)** LA in leaves of 2-, 3-, and 4-week-old plants (*Arabidopsis thaliana* wild-type [Col-0], *ugt76b1-1* and *UGT76B1-OE7*). Plants were grown on soil under short-day conditions (10 h photoperiod). Means ± SE (*n* = 4) are plotted. Asterisks indicate statistically significant differences in comparison to wild type (Col-0) (*ANOVA*), ^∗^*p*-value < 0.05, ^∗∗^*p*-value < 0.01. Dashed lines indicate the limit of detection (see section “Materials and Methods”).

UGT76B1 expression levels in the aboveground tissues are dependent on plant age (von Saint Paul et al., 2011). Therefore, we addressed the levels of ILA and LA during plant growth and development. Rosettes of 2-week-old *A. thaliana* plants grown on soil contain the highest level of ILA, which significantly decreased during plant growth (*p* < 0.0001; ANOVA) and was strongly dependent on genotypes (*p* < 0.0001; ANOVA) (**Figure 4A**). Interestingly, concentrations of ILA and LA of soil-grown plants were higher than those measured in seedlings grown on agar plates. Overall, much higher amounts of the aglycon ILA were found in the *ugt76b1* loss-of-function mutant. In contrast to ILA, LA content was not affected by the expression level of UGT76B1 throughout the growth stages examined (*p* > 0.05), but significantly increased in leaf extracts during plant growth (*p* < 0.0001; Kruskal–Wallis) (**Figure 4B**). Interestingly, the highest concentrations of LA were observed in 4-week-old leaves, when ILA was at the lowest level.

**FIGURE 5:**
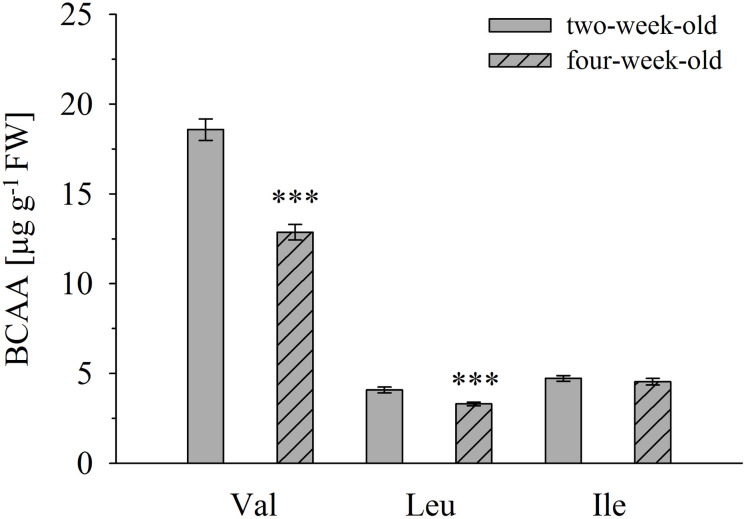
Branched-chain amino acid determination in *A. thaliana* accession Col-0 of 2- and 4-week-old plants. Amino acid concentrations were determined in leaves of 2- and 4-week-old seedlings. Means ± SE (*n* = 9–10) are plotted. Asterisks indicate statistically significant differences compared to 2-week-old plants (*t-*test). ^∗∗∗^*p* < 0.001.

**FIGURE 6:**
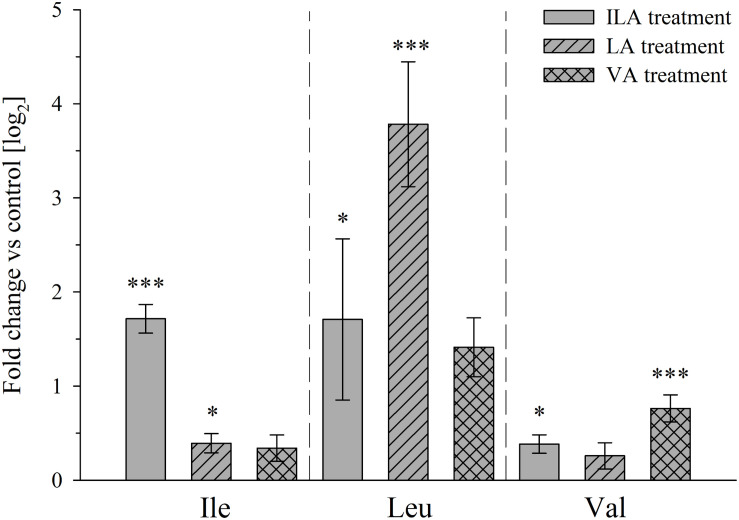
Branched-chain amino acid levels after exogenous application of 2-HAs to *A. thaliana* plants. Rosette leaves of 4-week-old *A. thaliana* Col-0 plants were harvested 24 h after spraying with 1 mM ILA, LA, or VA and extracted for BCAAs analysis. Means ± SE of log fold change of 2-HA treatments vs. mock are presented; (*n* = 8). Asterisks indicate statistically significant differences between treatment and mock (*ANOVA*), ^∗^*p*-value < 0.05, ^∗∗∗^*p*-value < 0.001. Note that the BCAA corresponding to a particular 2-HA is specifically induced indicating a metabolic relationship, while the respective other BCAAs are not affected or affected at a much lower extent.

Overall, no significant correlation between BCAA and 2-HA levels was observed, although we hypothesize that 2-HAs are linked to BCAA metabolism. The isoleucine levels remained unchanged between 2 and 4-week-old plants, whereas ILA abundance significantly decreased by plant age (**Figures 4A**, **5**). VA was not detectable (see above) despite the fact that valine was by far the most abundant BCAA. However, significantly decreasing levels of leucine from 2 to 4-week-old plants appeared in concert with increasing levels of LA (**Figures 4B**, **5**). Nevertheless, enzymatic reactions linking 2-HAs and BCAAs like in MSUD seem to exist in *Arabidopsis*, since a specific BCAA accumulates upon exogenous feeding of the corresponding 2-HA (**Figure 6**).

### ILA and LA Are Differently Affected by the Pathogen P. syringae

**FIGURE 7:**
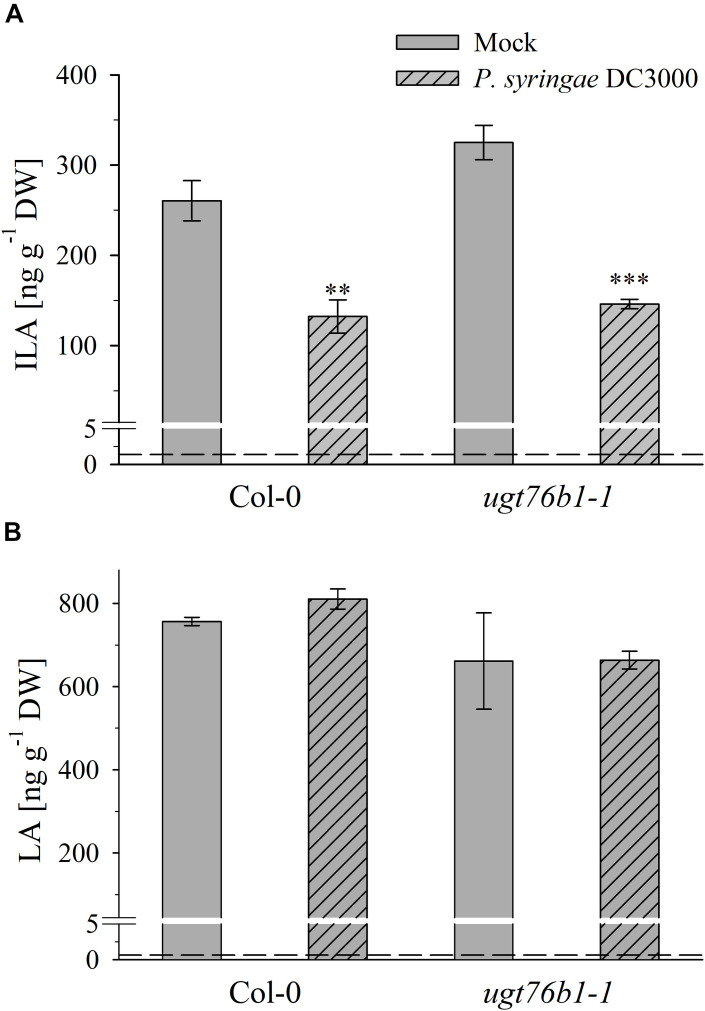
Isoleucic acid and LA abundance in response to *Pseudomonas syringae* virulent strain infection. Levels of **(A)** ILA and **(B)** LA aglyca 24 h post *P. syringae* DC3000 infection in leaves of *Arabidopsis* Col-0 and *ugt76b1-1* plants compared to mock controls. Means ± SE (*n* = 4) are plotted. Asterisks indicate statistically significant treatment effect compared to mock control (*t*-test), ^∗∗^*p* < 0.01, ^∗∗∗^*p* < 0.001. Dashed lines indicate the limit of detection (see section “Materials and Methods”).

Exogenously applied ILA stimulates plant defense (von Saint Paul et al., 2011). Therefore, we studied the impact of a (hemi-) biotrophic pathogen on the endogenous level of ILA. Infection with the virulent bacteria *P. syringae* DC3000 triggered a decrease of ILA abundance in Col-0 plants (**Figure 7A**). Interestingly, the same response could be observed in *ugt76b1-1* mutant plants (**Figure 7A**) indicating that the decrease of ILA in response to *P. syringae* DC3000 occurs independently from UGT76B1. In contrast to ILA, the isomeric LA was not affected by *P. syringae*, neither in wild-type nor in *ugt76b1-1* (**Figure 7B**). These responses were independent of the virulence of *P. syringae*; the avirulent strain *P. syringae avrRpm1* also triggered a decrease of ILA in wild-type plants, whereas LA levels remained unchanged (Supplementary Figure S5).

## Discussion

The 2-HA ILA has been discovered in its glucosylated form in *A. thaliana*. ILA glucoside formation was dependent on the activity of the small-molecule glucosyltransferase UGT76B1 *in planta* (von Saint Paul et al., 2011). Exogenous application of ILA activated SA-dependent defense relating it to plant pathogen response as a novel immune-modulating compound (von Saint Paul et al., 2011). This was in line with the *in vitro* activity of UGT76B1 glucosylating both ILA and SA, while ILA inhibited the glucosylation, i.e., inactivation, of SA by UGT76B1 (von Saint Paul et al., 2011; Noutoshi et al., 2012). However, the endogenous level of the aglycon ILA itself could not be assessed by the non-targeted metabolome analysis of von Saint Paul et al. (2011) due to lower instrumental sensitivities for small molecules (<150 a.m.u.). Previously, a targeted approach based on GC–MS has been used to quantify and identify 2-HAs in humans affected by MSUD (Jakobs et al., 1977; Podebrad et al., 1997; Chuang and Shih, 2001). MSUD is due to a genetic disorder of BCAA catabolism leading to the accumulation of 2-keto carboxylic acid catabolites and their reduced 2-HA derivatives ILA, LA and VA (Tanaka and Rosenberg, 1983; Mamer and Reimer, 1992; Chuang and Shih, 2001). These 2-HAs accumulate to high levels of 0.04–1.2 mM and even up to 30 mM in MSUD patients’ plasma and urine samples, respectively (Jakobs et al., 1977). Preliminary attempts could not detect ILA in crude plant extracts, indicating that levels of 2-HAs are low *in planta*. Therefore, we developed a more sensitive method for quantification of low abundant BCAA-related 2-HAs in plants. We could reach a limit of detection in the range of 10–27 fg mg^-1^ DW on spiked matrix calibration employing solid phase extraction followed by GC–MS analysis performed on a high-sensitivity instrument (allowing measurements at low ppt levels). Thereby, 2-HAs could be reliably detected in plant tissues.

**FIGURE 8:**
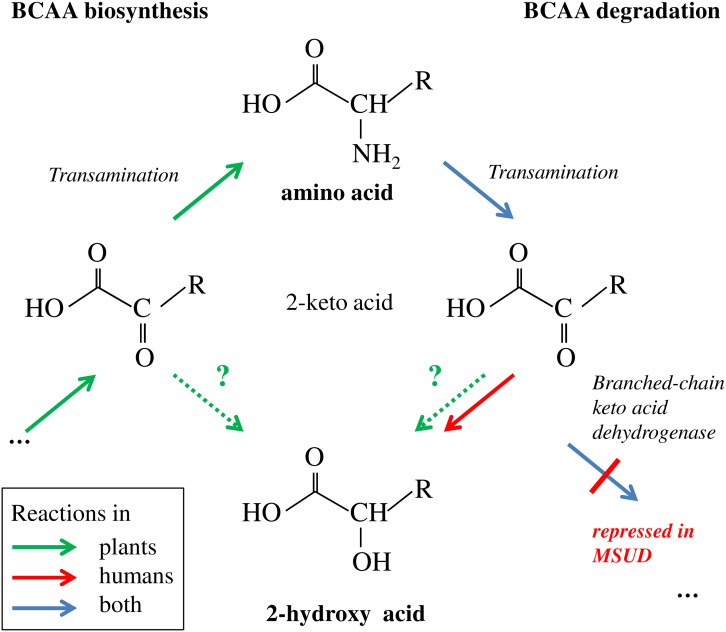
Branched-chain amino acid biosynthesis and degradation and putative link to 2-HA biogenesis. Plants, but not humans are able to synthesize BCAAs *de novo* involving transamination of the 2-keto acid precursor. Degradation in humans and plants involves the reverse transamination reaction leading to 2-keto acids. In MSUD the subsequent oxidative decarboxylation is repressed due to mutations in the branched-chain keto acid dehydrogenase complex; the accumulating 2-keto acids are in part reduced to 2-HAs (Chuang and Shih, 2001). In plants, the biosynthesis of BCAA-related 2-HAs is not known; however the corresponding 2-keto acids from biosynthetic or catabolic pathways are putative precursors (indicated by stippled arrows and question marks). R = CH(CH_3_)-C_2_H_5_ for Ile and derivatives, = CH_2_-CH(CH_3_)_2_ for Leu and derivatives, = CH(CH_3_)_2_ for Val and derivatives.

Branched-chain amino acid metabolism in plants and mammals differs in principle, since plants are able to synthesize them *de novo*, while BCAAs are essential amino acids in mammals (Binder, 2010). However, BCAA degradation proceeds in the same manner in plants and mammals (**Figure 8**). Although the link of the 2-HAs ILA, LA, and VA to BCAAs has not been clearly demonstrated in plants, it seems to be a plausible hypothesis that these compounds are related in plants to either the primary 2-keto acid degradation products of the amino acids like in MSUD or alternatively to the 2-keto acids as the ultimate precursors in BCAA biosynthesis (**Figures 6**, **8**). However, in contrast to 2-HAs in MSUD, ILA, and LA in *Arabidopsis* do not appear to be mere side-products of repressed BCAA degradation, but their formation is specifically regulated independent of the presence and relative amounts of the amino acids. This is evidenced by several observations. First, valine is clearly more abundant in *A. thaliana* than isoleucine and leucine; nevertheless, VA could not be detected irrespective of its sensitive detection in our analysis and its identification in other plant species. Second, ILA and LA differentially accumulate in response to pathogen infection. Third, ILA is declining when comparing 2 and 4-week-old *Arabidopsis* rosette leaves, while the corresponding amino acid Ile is not altered. On the other hand, LA, which strongly accumulates in 4-week-old leaves, was accompanied by an opposite change of Leu; this could indicate a link of declining Leu with raising LA levels. However, it has to be noted that 2-HA levels in *Arabidopsis* are several orders of magnitudes below the amounts of the corresponding BCAAs (**Figures 3**, **5**). In contrast, 2-HAs may reach almost similar plasma concentrations like BCAAs in MSUD patients (0.04 to 1.2 mM 2 HAs vs. 0.3 to 5 mM BCAAs; Jakobs et al., 1977; Tanaka and Rosenberg, 1983).

Several amino acids or amino acid-derived molecules are known to be relevant for plant defense response. The non-proteinogenic β-aminobutyric acid and the lysine catabolite pipecolic acid prime plant defense response (Návarová et al., 2012; Vogel-Adghough et al., 2013; Yang and Ludewig, 2014; Gao et al., 2015; Baccelli and Mauch-Mani, 2016; Bernsdorff et al., 2016). Pipecolic acid, which is formed after transamination of lysine, cyclization and reduction, is important for establishing systemic acquired resistance (Bernsdorff et al., 2016; Ding et al., 2016; Hartmann et al., 2017). The BCAA isoleucine is a critical moiety of the JA-Ile conjugate as the bioactive ligand in JA perception and signal transduction (Fonseca et al., 2009). However, the BCAAs Val, Leu, and Ile themselves were also linked to pathogen responses, since their abundance specifically increased upon *P. syringae* infection in *A. thaliana* and *N. tabacum* (Návarová et al., 2012; Vogel-Adghough et al., 2013). BCAAs are precursors for the biosynthesis of aliphatic glucosinolates, which are involved in plant defense against herbivores, fungi and biotrophic bacteria (Beekwilder et al., 2008; Bednarek et al., 2009; Sønderby et al., 2010; Fan et al., 2011; Zeier, 2013). In contrast to the enhanced BCAAs, the related 2-HAs LA as well as VA, which is also not detected in *A. thaliana* after infection, are not affected by pathogen infection, while ILA was lowered (**Figure 7**). This specific decrease of ILA after pathogen infection is independent of the virulence of the pathogenic bacteria. It could be functionally related to the inhibition of SA glucosylation by ILA found *in vitro* (Noutoshi et al., 2012). However, the averaged ILA concentrations are lower than SA levels in *A. thaliana*, thus an ILA-related inhibition might only be effective, if the ILA is not uniformly distributed in the plant tissue. Nevertheless, provided such an ILA-dependent inhibition of the SA glucosylation by UGT76B1, the reduction of ILA would release the inhibition of the SA-glucosylating activity of UGT76B1. An increased activity of UGT76B1 could be an attenuating measure of the SA response along with the transcriptional upregulation of *UGT76B1* after infection (von Saint Paul et al., 2011). Importantly, this scenario is independent of the glucosyltransferase UGT76B1 itself (**Figure 7**), indicating that ILA biogenesis is directly regulated as a response to pathogen infection. Nevertheless, the targeted metabolomic approach confirmed the presence of ILA and the impact of UGT76B1 on ILA levels *in vivo*. The loss-of-function *ugt76b1-1* line resulted in increased levels of free ILA, whereas a constitutive *UGT76B1* overexpression line led to a lowered ILA concentration. Surprisingly, the isomeric LA was not affected by different UGT76B1 levels *in vivo* (**Figures 3**, **4**), albeit LA was a substrate of UGT76B1 *in vitro* (**Table 1**). The biochemical ability of the enzyme is reasonable due to the highly similar structure of the aglyca and the frequently broad substrate specificity known of plant UGTs (Bowles et al., 2006); in fact, VA was also shown to be an *in vitro* substrate of UGT76B1 (von Saint Paul et al., 2011), although VA is not detected in *A. thaliana*. Thus, the *in vitro* activity of UGT76B1 toward LA may not be relevant *in vivo*, UGT76B1 and LA may not occur in the same cells or subcellular compartments, or the level of free LA is controlled to be stable and not influenced by UGT76B1.

Isoleucic acid and LA showed also opposite accumulation patterns during *Arabidopsis* development (see above). The highest content of ILA was found in 2-week-old *Arabidopsis* leaves, compared to 3- and 4-week-old plants. Conversely, LA content increased during this time span. On the other hand, the amounts of the related amino acids Ile and Leu were not altered accordingly. Thus, it appears that the pools of ILA and LA are specifically regulated during growth and not linked to the BCCA levels suggesting a distinct, yet unexplored role of these two compounds in plant development.

## Conclusion

The improved sensitive method to quantify 2-HAs related to BCAAs revealed their differential accumulation and distinct relation to plant development and defense. All three 2-HAs were identified in plants at different levels. However only ILA was detected in all plants examined, which suggests a special role for this compound involved in defense modulation in *A. thaliana*.

## Author Contributions

RM, VvSP, J-PS, and AG developed the concept and 2-HAs analytics. RM, BL, WZ, and AG performed experiments to quantify 2-HAs. BG and BL performed *in vitro* analyses using recombinant UGT76B1. M-RH analyzed amino acid levels. RM, AG, and AS evaluated the data and wrote the manuscript.

## Conflict of Interest Statement

The authors declare that the research was conducted in the absence of any commercial or financial relationships that could be construed as a potential conflict of interest.

## Footnotes

1 http://kanaya.naist.jp/KNApSAcK/

2 http://www.r-project.org

## Abbreviations

BCAA: branched-chain amino acid
BSTFA: *N, O-bis* (trimethylsilyl)-trifluoroacetamide
DW: dry weight
ETI: effector-triggered immunity
2-HA: 2-hydroxy (carboxylic) acid
ILA: isoleucic acid
IS: internal standard
JA: jasmonic acid
LA: leucic acid
MSUD: maple syrup urine disease
PAMP: pathogen-associated molecular pattern
PPFD: photosynthetic photon flux density
PTI: pathogen-triggered immunity
SA: salicylic acid
TMCS: trimethylchlorosilane
VA: valic acid
