# Corrigendum: The Defense-Related Isoleucic Acid Differentially Accumulates in *Arabidopsis* Among Branched-Chain Amino Acid-Related 2-Hydroxy Carboxylic Acids

**DOI:** 10.3389/fpls.2020.01088

**Published:** 2020-08-12

**Authors:** Rafał P. Maksym, Andrea Ghirardo, Wei Zhang, Veronica von Saint Paul, Birgit Lange, Birgit Geist, Mohammad-Reza Hajirezaei, Jörg-Peter Schnitzler, Anton R. Schäffner

**Affiliations:** ^1^Institute of Biochemical Plant Pathology, Helmholtz Zentrum München, Munich, Germany; ^2^Research Unit for Environmental Simulation, Helmholtz Zentrum München, Munich, Germany; ^3^Molecular Plant Nutrition, Leibniz-Institute for Plant Genetics and Crop Plant Research, Gatersleben, Germany

**Keywords:** isoleucic acid, branched-chain amino acids, *Arabidopsis thaliana*, plant pathogen defense, UGT76B1, *Pseudomonas syringae*, GC–MS, MSUD

## Figure Legend

In the original article, there was a mistake in the legend for **Figure 6** and **Figure 7** as published.

The numbering for **Figure 6** was mixed with **Figure 7** and not in agreement with the text: this mistake was introduced during proof stage, since the editing changed both legend and figure number. In addition, DC3000 should not be in italics. The correct numbering of **Figures 6** and **7** appear below.

**FIGURE 6 | BCAA levels after exogenous application of 2-HAs to *A. thaliana* plants**

**FIGURE 7 | ILA and LA abundance in response to *P. syringae* virulent strain infection**.

## Error in Figure

In the original article, there was a mistake in **Figures 2**–**4**, and **6** as published. Due to the incorrect calculation of 2-HA level (a factor 50 was missed), all figures showing such measurements have to be replaced with the corrected y axis and limit of detection. The corrected **Figures 2**–**4**, and **7** appear below.

**Figure 2:**
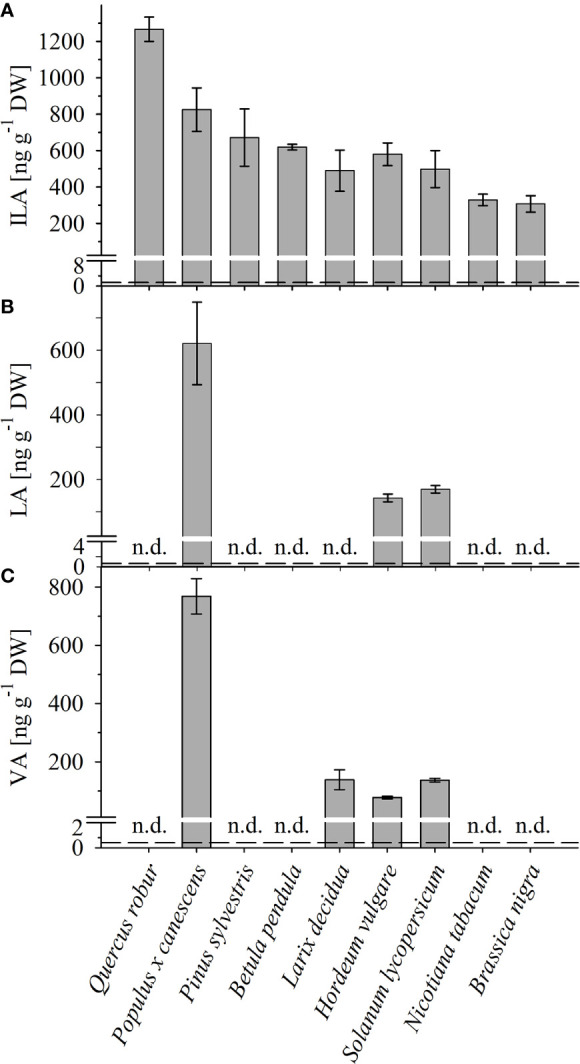
Detection of ILA, LA, VA in different plant species. Abundance of **(A)** ILA, **(B)** LA, and **(C)** VA in leaf extracts of monocot (*Hordeum vulgare*) and dicot crops (*Solanum lycopersicum, Nicotiana tabacum, Brassica nigra*), as well as of broadleaf (*Quercus robur, Populus x canescens, Betula pendula*) and coniferous (*Pinus sylvestris, Larix decidua*) trees. Means ± SE (n = 3–4) are plotted; n.d., not detected. Dashed lines indicate the limit of detection (see section “Materials and Methods”).

**Figure 3:**
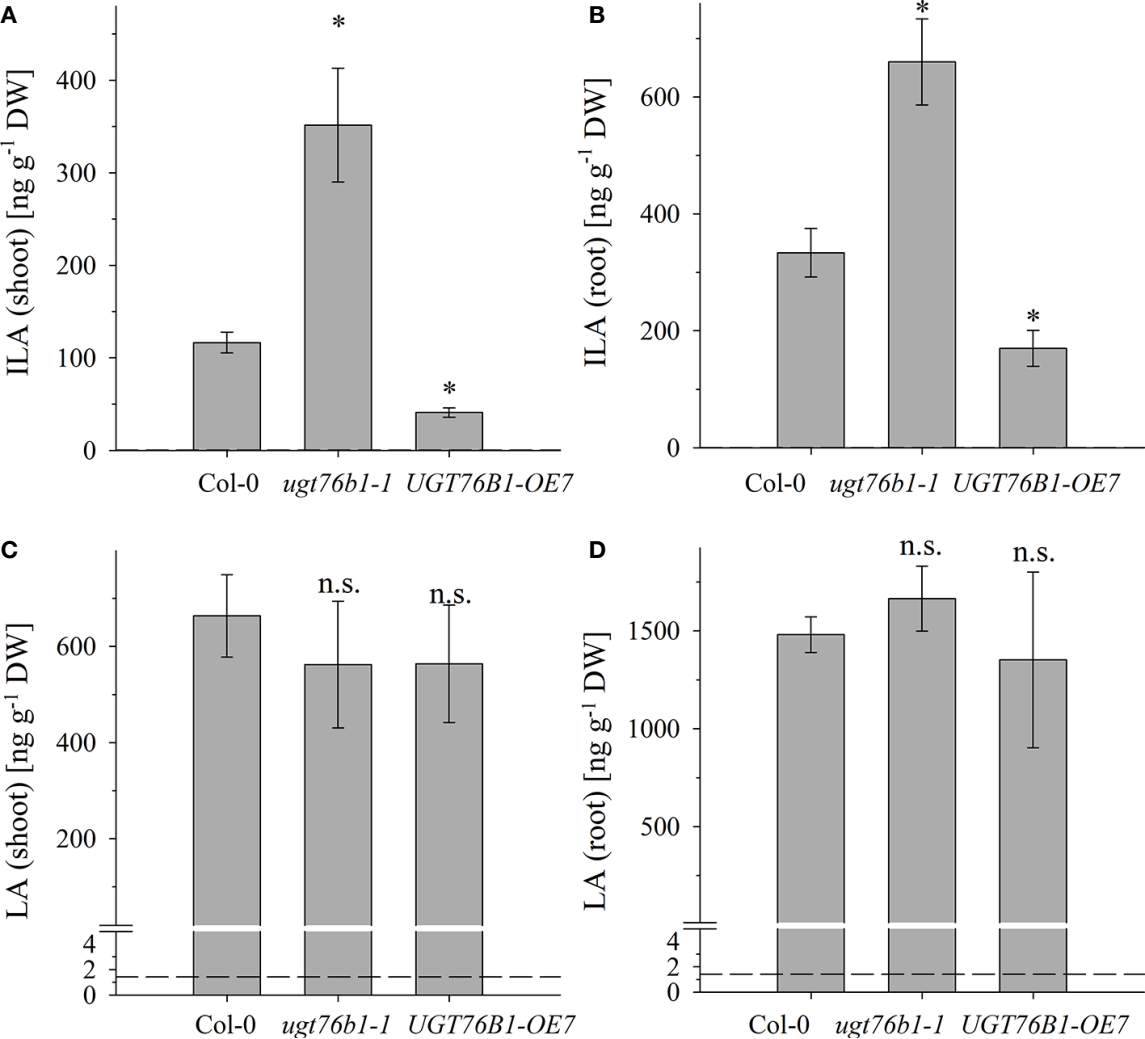
The abundance of ILA and LA in above- and below-ground tissues of Col-0, *ugt76b1-1* and *UGT76B1-OE7*. Levels of **(A, B)** ILA and **(C, D)** LA using **(A, C)** shoot and **(B, D)** root of 3-week-old Col-0, *ugt76b1-1* and *UGT76B1-OE7* plants grown on Gelrite plates in short-day conditions (10 h photoperiod). Means ± SE (n = 4) are plotted. Asterisks indicate statistically signiﬁcant differences in comparison to wild type (Col-0) (ANOVA), *p-value < 0.05; n.s., not significant. Dashed lines indicate the limit of detection (see section “Materials and Methods”).

**Figure 4:**
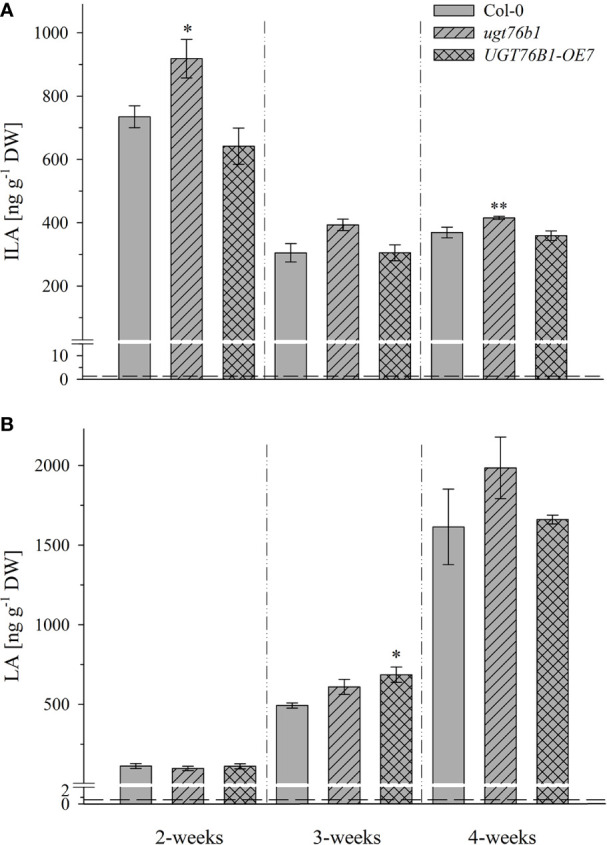
Isoleucic acid and LA abundance in different developmental stages. Levels of **(A)** ILA and **(B)** LA in leaves of 2-, 3-, and 4-week-old plants (*Arabidopsis thaliana* wild-type [Col-0], *ugt76b1-1* and UGT76B1-OE7). Plants were grown on soil under short-day conditions (10 h photoperiod). Means ± SE (n = 4) are plotted. Asterisks indicate statistically signiﬁcant differences in comparison to wild type (Col-0) (ANOVA), *p-value < 0.05, **p-value < 0.01. Dashed lines indicate the limit of detection (see section “Materials and Methods”).

**Figure 7:**
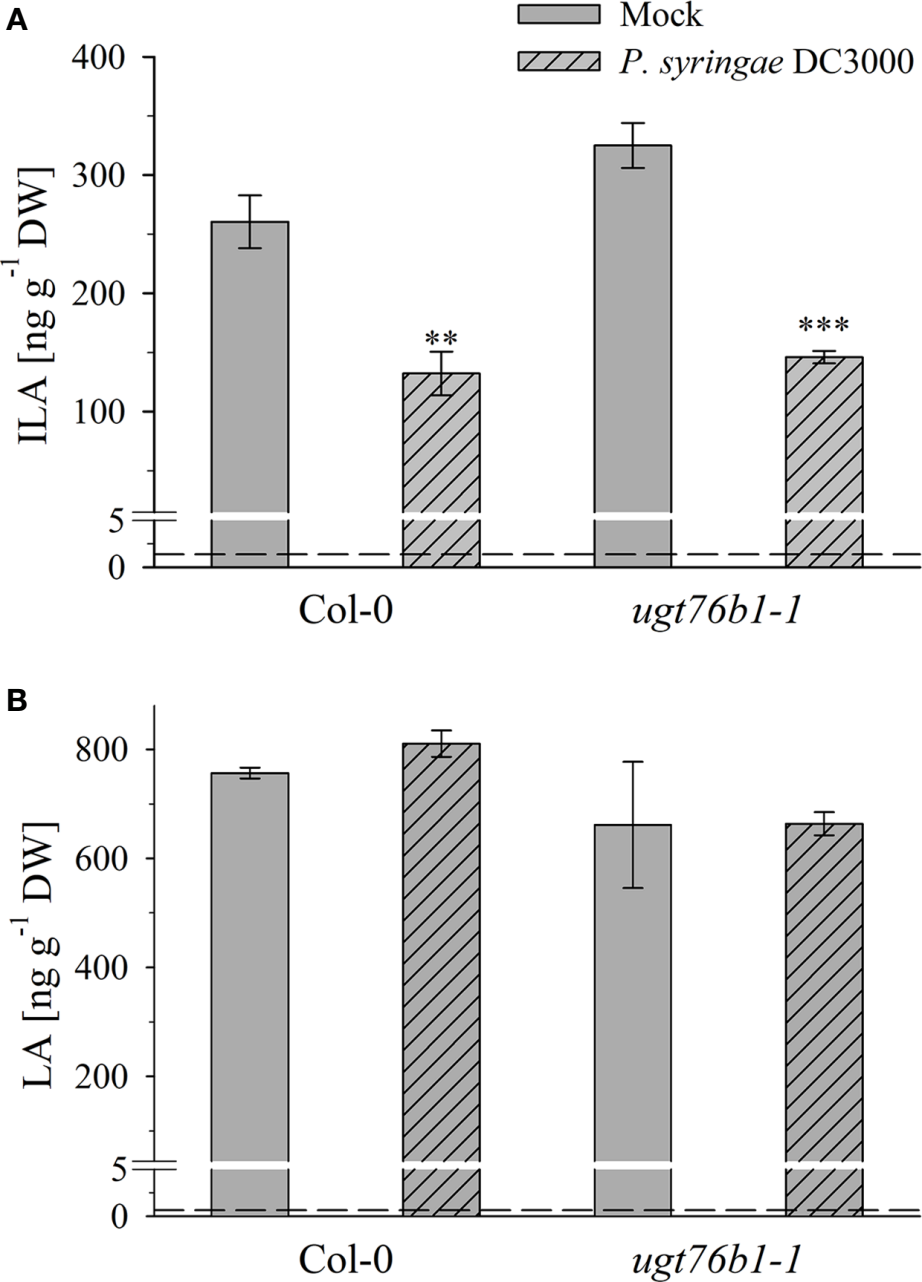
Isoleucic acid and LA abundance in response to *Pseudomonas syringae* virulent strain infection. Levels of **(A)** ILA and **(B)** LA aglyca 24 h post *P. syringae* DC3000 infection in leaves of Arabidopsis Col-0 and *ugt76b1-1* plants compared to mock controls. Means ± SE (n = 4) are plotted. Asterisks indicate statistically signiﬁcant treatment effect compared to mock control (t-test), ^∗∗^p < 0.01, ^∗∗∗^p < 0.001. Dashed lines indicate the limit of detection (see section “Materials and Methods”).

## Text Correction

### Issue 1

In the original article, there was an error in the following section: ***Materials and Methods*, *Determination of ILA, LA and VA by GC-MS***, ***Paragraph 2***:. The description of the addition of standards was ambiguous and not correct; this also lead to a wrong calculation of the final 2-HA concentrations, which all have to be multiplied by the factor 50. This error affects the relative content of 2-HA, e.g., in comparison to other plant metabolites like BCAAs or salicylic acid. The correct paragraph 2 into ***Materials and Methods*, *Determination of ILA, LA and VA by GC-MS***, ***appears below***:

Qualitative and quantitative analyses of VA, LA and ILA were performed by gas chromatography-mass spectrometry (GC-MS). Samples were analyzed with a thermo-desorption unit (Gerstel, Mülheim an der Ruhr, Germany) coupled to a GC-MS instrument (GC type: 7890; MS type: 5975C, both Agilent Technologies, Palo Alto, CA, USA). The thermo-desorption unit was used as injector for the conversion of the sample from liquid to gas-phase. GC-MS was run as follows: One µL of sample was injected into the thermo-desorption unit in a dedicated glass tube containing the glass insert for liquid injection (both from Gerstel, Mülheim an der Ruhr, Germany). Prior to each analysis, tubes and inserts were accurately cleaned with acetone, methanol and water, separately used in ultrasonic bath for 30 min each, and kept in hexane solution overnight. Immediately before analysis, tubes were baked out in oven at 400°C for 1 h under ~80 mL min^-1^ N_2_ (5.0 gas purity) flow. Samples were vaporized by quickly rising the temperature from 40 to 270°C at a rate of 360°C min^-1^ and holding for 0.5 min. The compounds were refocused using a Cryo Injection System (Gerstel, Mülheim an der Ruhr, Germany) at -50°C, then desorbed and injected in splitless mode by rising the temperature to 250°C at a rate of 12°C sec^-1^ and hold for 1.5 min, followed by ramping at 12°C sec^-1^ to 275°C and holding for 2 min. Separation was achieved by using the Agilent J&W HP-5ms GC column (30 m x 250 µm x 0.25 µm) with 1 mL min^-1^ constant flow rate of He, and a temperature program of 90°C for 4 min, followed by ramping at 2°C min^-1^ to 120°C and holding for 0 min, then 100°C min^-1^ to 300°C and holding for 5 min. Identification of VA, LA, ILA, and the two IS (2-hydroxyhexanoic acid and 4-nitrophenol) were achieved by spectra and retention time comparison of pure standards. The quantification was obtained by means of a calibration curve obtained from pure standards. MS spectra were parallelly acquired in total ion current and in selective ion monitoring modes. Scans of total ion current were performed in the range of 35-300 mass-to-charge ratios (m/z) (threshold: 150; 7.76 scan sec^-1^). Selective ion monitoring parameters were as follows, VA: start time: 6.2 min, ion: 145.0 m/z, dwell: 150 ms; LA: start time: 8.5 min, ion: 159.0 m/z, dwell: 150 ms; ILA: start time: 11.5 min, ion: 159.0 m/z, dwell: 150 ms; 2-hydroxyhexanoic acid: start time: 13.9 min, ion: 173.1 m/z, dwell: 100 ms; 4-nitrophenol: start time: 16.0 min, ion: 196.1 m/z, dwell: 25 ms. MS detector was kept off until 6.20 min and switched off after 20.65 min until the end of the run. The calibration was achieved by adding standards into a mixture of *Arabidopsis* plant extracts, to take into account potentially occurring matrix effects. The respective 2-hydroxy acid standards were directly added into 1 mL of the pooled 70/30% methanol/water plant extract. After all preparative steps, the final standard concentrations of the BSTFA solution used for GCMS injection were of 0, 0.05, 0.1, 0.15, 0.2, 0.25, 0.3, 0.5, 1, 5, 10 ng µL^-1^. Each concentration of ILA contained the same fix concentration of IS (50 mg L^-1^). Calibration samples were treated in exactly the same way as the sample preparation explained above (i.e. passing through the SPE columns). Data were background corrected using the mean value obtained from measuring the plant extract with the addition of the standard solution lacking ILA to correct for the basal levels of ILA present in the pooled plant material. Standards were prepared independently in triplicate, and each concentration was measured twice. The two technical replicates were averaged and their means were further used for the calculation of response factors. To consider uncertainties of standard preparation, the quantification of 2-HAs were based on three independently created serial dilutions. The resulting MS signal responses were found to be linear (R^2^> 0.9999) with an increasing standard concentration. Response factors of VA and LA were calculated based on the matrix-dependent calibration curve of ILA assuming that the matrix effects occurred at the same extent for VA, LA and ILA: serial dilutions of pure standards (0-100 mg L^-1^) of ILA, VA and LA were measured in parallel and the ratios of VA/ILA and LA/ILA were applied to the matrix-dependent response factor of ILA. Data were always normalized to IS values of 4-nitrophenol. Samples showing an inconsistent ratio of the two IS were discarded from the analysis. Limits of detection were calculated using 2 sigma (σ) and were ranging between 1.35-64.5 (ILA), 2.25-11.45 (VA), 0.6-1.45 (LA) ng g^-1^ dry weight (DW), referred to *A. thaliana* plant material. The limits of quantification (LOQ) were set to three times of their respective limits of detection.

### Issue 2

In the original article, there was an error in the Results section, **Determination of BCAA-related 2-HAs in Plants**, **Paragraph 1**.

The numbers of the limit of detection have to be adapted. The correct paragraph 1 appears below.

To identify and quantify the abundance of valic acid (VA, 2-hydroxy-isovaleric acid), leucic acid (LA, 2-hydroxyisocaproic acid) and isoleucic acid (ILA, 2-hydroxy-3-methyl-valeric acid) in plants, we developed a sensitive method based on derivatization of these molecules by silylation and GC-MS analysis (**Figure 1**; **Supplementary Data Figure S1**). With this method it was possible to detect low amounts of the BCAA-related compounds. The limits of detection were ranging between 1.35-64.5 (ILA), 2.25-11.45 (VA), 0.6-1.45 (LA) ng g^-1^ DW depending on instrument performance and background noise. To demonstrate the general versatility of the procedure different plant species were examined for their content in ILA, LA and VA. ILA could be ubiquitously detected in all examined plant species including monocotyledonous and dicotyledonous plants, herbaceous and woody plants, whereas VA and LA were found only in some species ( **Figure 2**; **Supplementary Data Figures S2, S3**). All three 2-HAs could be detected in *Populus x canescens*, *Hordeum vulgare* and *Solanum lycopersicum* (**Figure 1**). In the model plant *A. thaliana* only LA and ILA were present in the extracts (**Figure 3**, **Supplementary Data Figure S3**).

### Issue 3

In the original article, there was an error in the *Discussion* section, Paragraph 1, as a consequence of the adapted limit of detection. The correct paragraph appears below.

The 2-HA ILA has been discovered in its glucosylated form in *Arabidopsis thaliana*. ILA glucoside formation was dependent on the activity of the small-molecule glucosyltransferase UGT76B1 *in planta* (von Saint Paul et al., 2011). Exogenous application of ILA activated SA-dependent defense relating it to plant pathogen response as a novel immune-modulating compound (von Saint Paul et al., 2011). This was in line with the *in vitro* activity of UGT76B1 glucosylating both ILA and SA, whereas ILA inhibited the glucosylation, i.e. inactivation, of SA by UGT76B1 (von Saint Paul et al., 2011; Noutoshi et al., 2012). However, the endogenous level of the aglycon ILA itself could not be assessed by the non-targeted metabolome analysis of von Saint Paul et al. (2011) due to lower instrumental sensitivities for small molecules (<150 a.m.u.). Previously, a targeted approach based on GC-MS has been used to quantify and identify 2-HAs in humans affected by MSUD (Jakobs et al., 1977; Jakobs et al., 1977; Chuang and Shih, 2001). MSUD is due to a genetic disorder of BCAA catabolism leading to the accumulation of 2-keto carboxylic acid catabolites and their reduced 2-HA derivatives ILA, LA and VA (Mamer and Reimer, 1992; Tanaka and Rosenberg, 1983; Chuang and Shih, 2001). These 2-HAs accumulate to high levels of 0.04 – 1.2 mM and even up to 30 mM in MSUD patients' plasma and urine samples, respectively (Jakobs et al., 1977). Preliminary attempts could not detect ILA in crude plant extracts, indicating that levels of 2-HAs are low *in planta*. Therefore, we developed a more sensitive method for quantification of low abundant BCAA-related 2-HAs in plants. We could reach a limit of detection in the range of 0.6 (LA) - 1.35 (ILA) ng g^-1^ DW on spiked matrix calibration employing solid phase extraction followed by GC-MS analysis performed on a high-sensitivity instrument (allowing measurements at low ppt levels). Thereby, 2-HAs could be reliably detected in plant tissues.

### Issue 4

In the original article, there was an error in **the *Discussion* section, *Paragraph 3*.**

The relation of 2-HA levels to those of BCAAs and salicylic acid, which are addressed in the discussion, has to be changed and adapted, since the calculation of 2-HA levels had to be corrected leading to 50fold higher levels: The higher, correct levels of 2-HA are important when comparing them to the signaling molecule salicylic acid and render the results more significant, obviating remark on its low concentration. The correct paragraph 3 appears below.

Several amino acids or amino acid-derived molecules are known to be relevant for plant defense response. The non-proteinogenic β-aminobutyric acid and the lysine catabolite pipecolic acid prime plant defense response (Návarová et al., 2012; Vogel-Adghough et al., 2013; Yang and Ludewig, 2014; Gao et al., 2015; Baccelli and Mauch-Mani, 2016; Bernsdorff et al., 2016). Pipecolic acid, which is formed after transamination of lysine, cyclization and reduction, is important for establishing systemic acquired resistance (Bernsdorff et al., 2016; Ding et al., 2016; Hartmann et al., 2017). The BCAA isoleucine is a critical moiety of the JA-Ile conjugate as the bioactive ligand in JA perception and signal transduction (Fonseca et al., 2009). However, the BCAAs Val, Leu and Ile themselves were also linked to pathogen responses, since their abundance specifically increased upon *P. syringae* infection in *A. thaliana* and *N. tabacum* (Návarová et al., 2012; Vogel-Adghough et al., 2013). BCAAs are precursors for the biosynthesis of aliphatic glucosinolates, which are involved in plant defense against herbivores, fungi and biotrophic bacteria (Beekwilder et al., 2008, Bednarek et al., 2009; Sønderby et al., 2010; Fan et al., 2011; Zeier, 2013). In contrast to the enhanced BCAAs, the related 2-HAs LA as well as VA, which is also not detected in *A. thaliana* after infection, are not affected by pathogen infection, while ILA was lowered (**Figure 7**). This specific decrease of ILA after pathogen infection is independent of the virulence of the pathogenic bacteria. It could be functionally related to the inhibition of SA glucosylation by ILA found *in vitro* (Noutoshi et al., 2012). The local levels of ILA and SA in specific cells are not known, but their average levels are comparable, making such a scenario possible *in vivo* as well. Thus, the reduction of ILA would release the inhibition of the SA-glucosylating activity of UGT76B1. An increased activity of UGT76B1 could be an attenuating measure of the SA response along with the transcriptional upregulation of *UGT76B1* after infection (von Saint Paul et al., 2011). Importantly, this scenario is independent of the glucosyltransferase UGT76B1 itself (**Figure 7**), indicating that ILA biogenesis is directly regulated as a response to pathogen infection. Nevertheless, the targeted metabolomic approach confirmed the presence of ILA and the impact of UGT76B1 on ILA levels *in vivo*. The loss-of-function *ugt76b1-1* line resulted in increased levels of free ILA, whereas a constitutive *UGT76B1* overexpression line led to a lowered ILA concentration. Surprisingly, the isomeric LA was not affected by different UGT76B1 levels *in vivo* (**Figures 3** and **4**), albeit LA was a substrate of UGT76B1 *in vitro* (**Table 1**). The biochemical ability of the enzyme is reasonable due to the highly similar structure of the aglyca and the frequently broad substrate specificity known of plant UGTs (Bowles et al., 2006); in fact, VA was also shown to be an *in vitro* substrate of UGT76B1 (von Saint Paul et al., 2011), although VA is not detected in *A. thaliana*. Thus, the *in vitro* activity of UGT76B1 towards LA may not be relevant *in vivo*, UGT76B1 and LA may not occur in the same cells or subcellular compartments, or the level of free LA is controlled to be stable and not influenced by UGT76B1.

The authors apologize for these errors and state that they do not change the scientific conclusions of the article in any way. The original article has been updated.

