# Supplementary material for: The Defense-Related Isoleucic Acid Differentially Accumulates in Arabidopsis Among Branched-Chain Amino Acid-Related 2-Hydroxy Carboxylic Acids
Source: Front Plant Sci. 2018 Jun 8;9:766. doi: 10.3389/fpls.2018.00766 (PMC6002512; doi:10.3389/fpls.2018.00766)
Supplement: Supplementary file 1 [file Presentation_1.PDF]

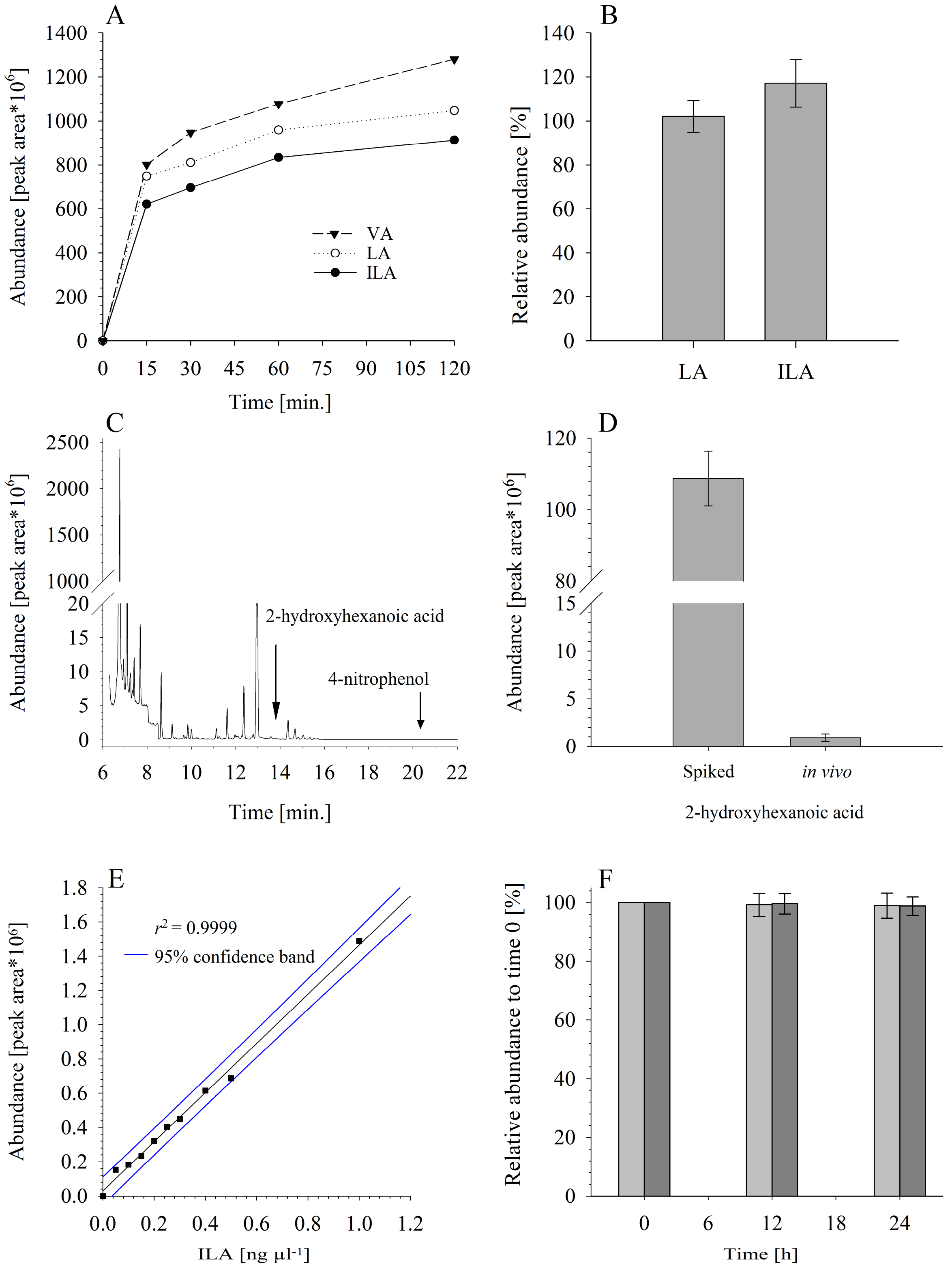
A
Abundance [peak area*10^6]
Time [min.]
VA
LA
ILA
B
Relative abundance [%]
LA
ILA
C
Abundance [peak area*10^6]
2-hydroxyhexanoic acid
4-nitrophenol
Time [min.]
D
Abundance [peak area*10^6]
Spiked
in vivo
2-hydroxyhexanoic acid
E
Abundance [peak area*10^6]
r² = 0.9999
95% confidence band
ILA [ng µl-1]
F
Relative abundance to time 0 [%]
Time [h]

SUPPLEMENTARY DATA FIGURE S1 | Optimization of GC-MS based method for 2-HA derivatives of BCAAs. (**A**) Dependence of the abundances of derivatized VA, LA, ILA (100 ng μl^{-1}) on the time of incubation for BSTFA silylation at 60 °C. (**B**) Impact of freeze-drying of plant material on LA and ILA quantification. Plant material was harvested, grinded in liquid N2, then split into equal batches; one was directly used for metabolite extraction and the second one was lyophilized prior to metabolite extraction. The levels of LA and ILA in extracts from lyophilized material relative to those in extracts from fresh material are depicted. Means ± s.e. (n = 3-4). (**C**) Typical Selective Ion Monitoring chromatogram of *A. thaliana* extract without spiking two internal standards (IS). Arrows indicate the expected retention time of the IS and show that no major peaks from plant extracts are overlapping with the IS. (**D**) Endogenous levels of 2-hydroxyhexanoic acid in comparison to the IS concentration used. Means ± s.e. (n = 3). (**E**) Typical linear relationship between injection of ILA standard and MS abundance signals obtained during a dilution series (calibration) procedure. (**F**) Stability of the derivatized form of ILA (light grey) and LA (dark grey) from leaf extracts of *A. thaliana*. Samples were kept over a period of 24 h at room temperature. Means ± s.e. (n = 20).

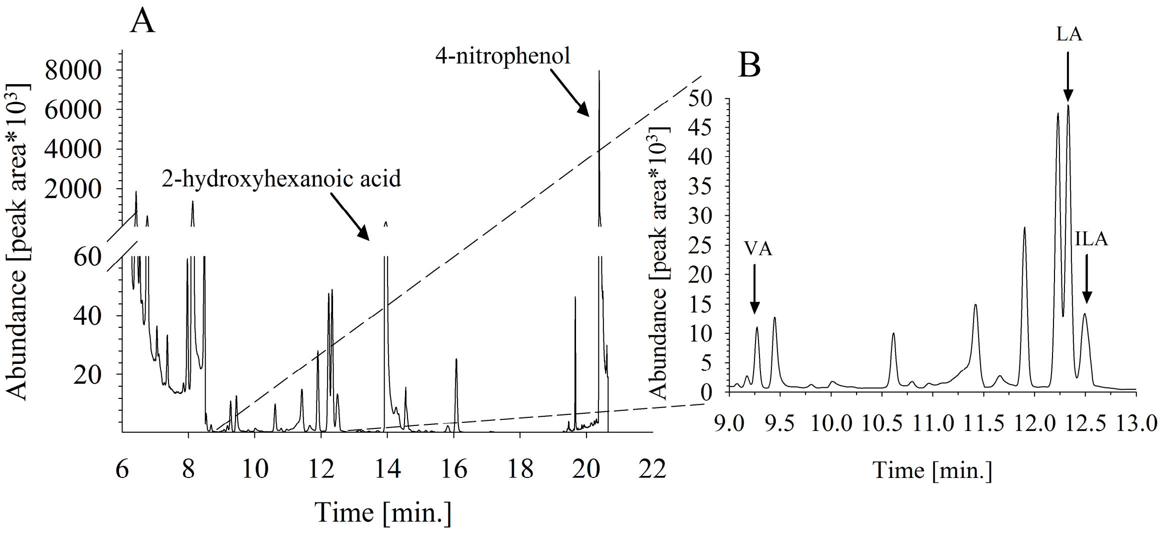

SUPPLEMENTARY DATA FIGURE S2 | Detection of BCAA derivatives in *Populus* extracts. (**A**) Selective Ion Monitoring (SIM) chromatogram of VA, LA, ILA, 2-hydroxyhexanoic acid and 4-nitrophenol after silylation with BSTFA. (**B**) Enlargement of the chromatogram eluting VA, LA and ILA. Retention times (min) and ions (m/z) monitored: 6.2 min, 147.0 m/z; 8.5 min, 145.0 m/z; 11.5 min, 159.0 m/z; 13.9 min, 173.1 m/z; 16.0 min, 196.1 m/z.

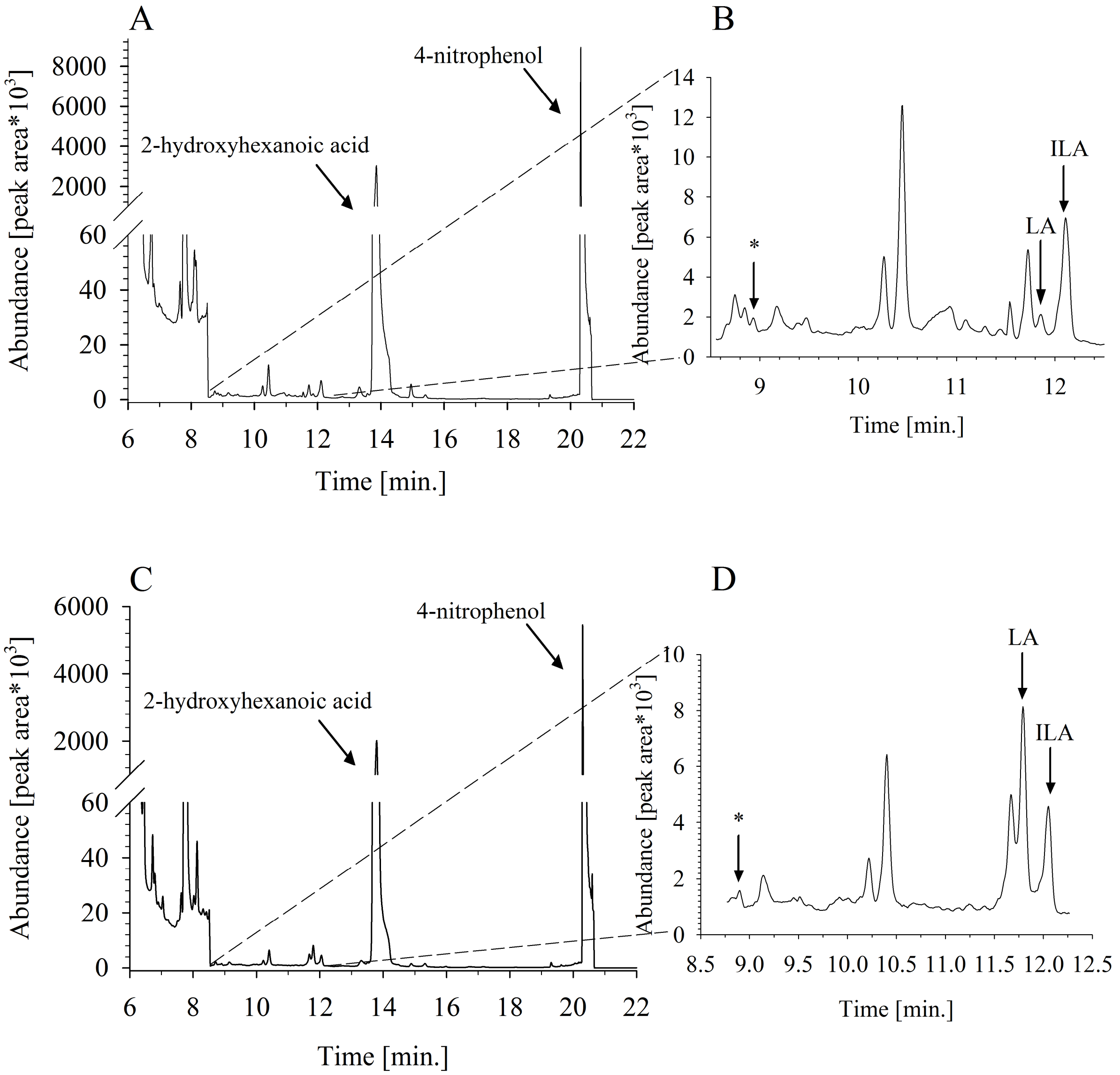

SUPPLEMENTARY DATA FIGURE S3 | Detection of BCAA derivatives in *A. thaliana* extracts. Selective Ion Monitoring chromatogram of LA, ILA, 2-hydroxyhexanoic acid and 4-nitrophenol after silylation with BSTFA in leaf extracts of (**A**) two-week-old and (**C**) four-week-old plants. (**B**, **D**) Enlargement of the chromatogram regions where LA and ILA elute. (*) indicates the expected position of the VA peak. Retention times (min) and ions (m/z) monitored: 6.2 min, 147.0 m/z; 8.5 min, 145.0 m/z; 11.5 min, 159.0 m/z; 13.9 min, 173.1 m/z; 16.0 min, 196.1 m/z.

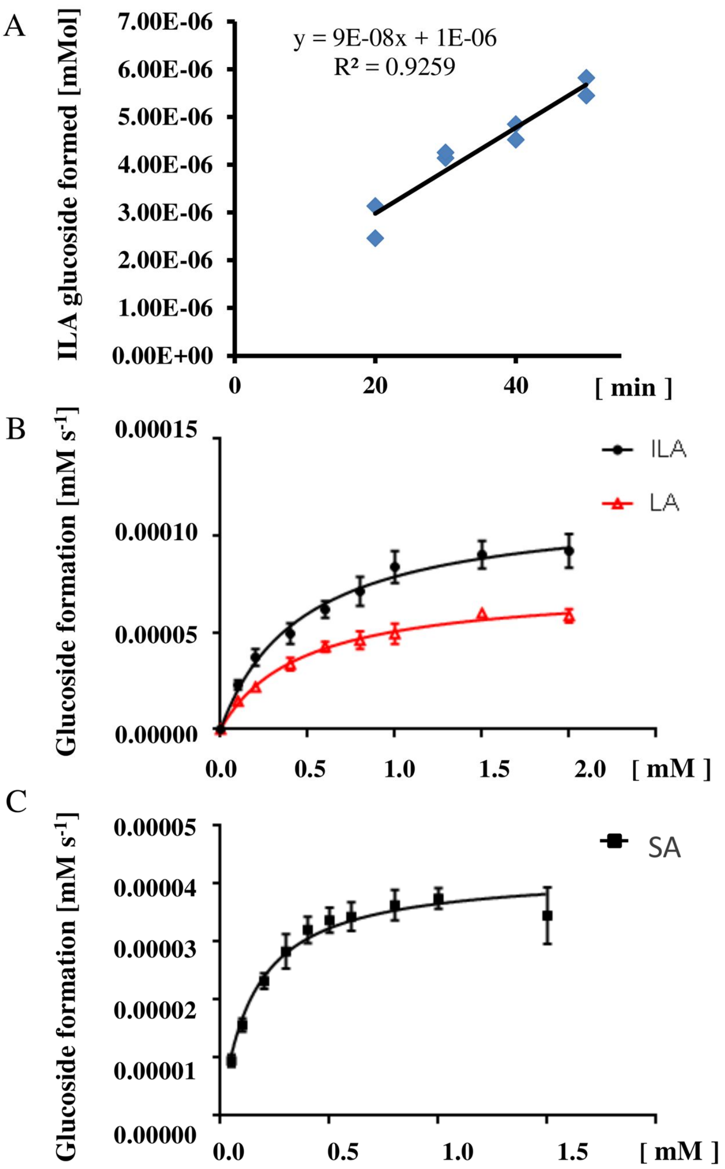

SUPPLEMENTARY DATA FIGURE S4 | Enzymatic parameters of recombinant UGT76B1. (**A**) Time-dependence of ILA glucoside formation in a standard reaction using 0.5 µg recombinant UGT76B1 fusion protein with 0.5 mM ILA and 2 mM ^{14}C-labeled UDP-glucose (Materials and Methods). (**B**) Concentration dependence of ILA and LA glucoside formation to determine K_m and k_{cat} values according to Michaelis-Menten kinetics. Plot is exported from GraphPadPrism (Materials and Methods). Reactions have been performed at least in triplicates with a total of at least 34 data points; mean values ± s.e. are shown for the individual concentrations. (**C**) Concentration dependence of SA glucoside formation to determine K_m and k_{cat} values. Plot is exported from GraphPadPrism (Materials and Methods). Reactions have been performed in triplicates with a total of 30 data points; mean values ± s.e. are shown for the individual concentrations.

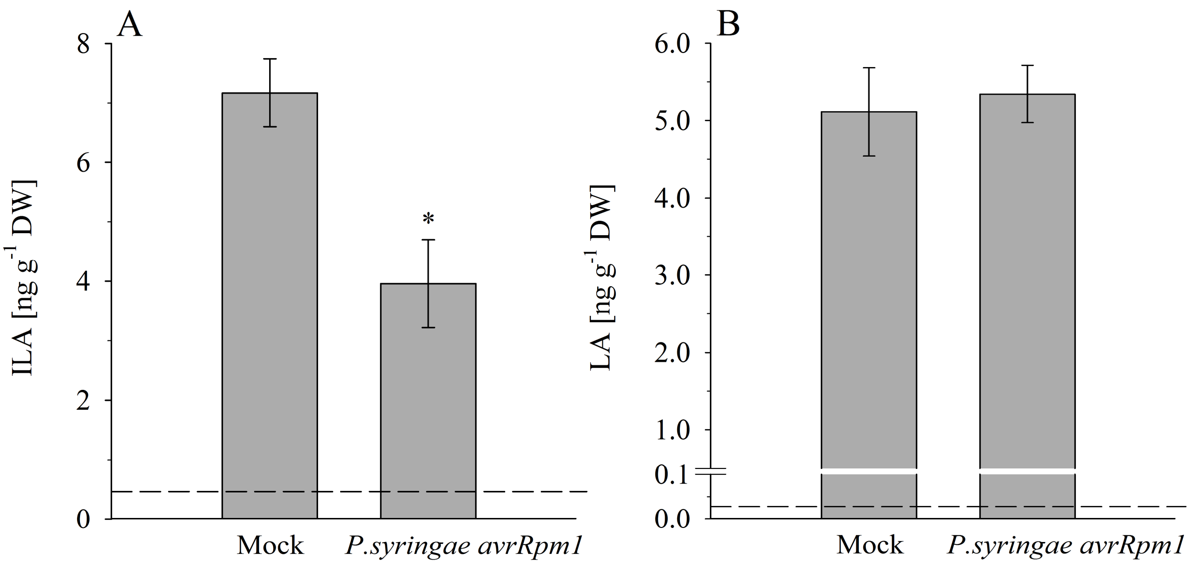

SUPPLEMENTARY DATA FIGURE S5 | ILA and LA abundance in response to *P. syringae* avirulent strain infection. Levels of (**A**) ILA and (**B**) LA 24 hours post *P. syringae avrRptm1* infection in four-week-old Col-0 plants. Means ± s.e. (n = 4). Asterisks indicate statistically significant treatment effect compared to mock control (*t*-test); *, $p < 0.05$. Dashed lines indicate the limit of detection.
